# Successful Treatment of Aplastic Anemia With Eltrombopag During Pregnancy: A Short Report

**DOI:** 10.1002/jha2.70244

**Published:** 2026-03-11

**Authors:** Sandra M. Frey, Ferras Alashkar, H. Christian Reinhardt, Alexander Röth

**Affiliations:** ^1^ Department of Hematology and Stem Cell Transplantation, West German Cancer Center University Hospital Essen, University Duisburg‐Essen Essen Germany

**Keywords:** aplastic anemia, eltrombopag, pregnancy

## Abstract

**Introduction:**

Aplastic anemia (AA) is a rare bone marrow failure syndrome with pancytopenia, mainly due to immune‐mediated stem cell destruction. First‐line therapy for acquired severe AA ≥ 50 years/non‐severe AA (NSAA) requiring treatment is immunosuppressive therapy with horse anti‐thymocyte globulin, cyclosporine A (CSA), and eltrombopag (EPAG). In pregnancy, cytopenia may worsen, while therapeutic options are limited.

**Results:**

We report the first case of a pregnant patient with NSAA/PNH receiving full‐dose EPAG (150 mg/d). Counts remained stable, delivery was uneventful, and the child was healthy. Postpartum, EPAG was discontinued, CSA tapered, and transfusion independence achieved.

**Conclusion:**

EPAG may represent a feasible option in selected pregnancies.

## 1 Introduction

Aplastic anemia (AA) is a rare, life‐threatening bone marrow failure disorder, characterized by bi‐ or pancytopenia and a hypocellular bone marrow in the absence of pathological infiltrates or fibrosis. It is mainly caused by immune‐mediated destruction of hematopoietic stem cells. Up to 70% of patients diagnosed with acquired AA also exhibit hematopoietic cells deficient in glycosylphosphatidylinositol‐anchored proteins, namely paroxysmal nocturnal hemoglobinuria (PNH)–type blood cells [1]. First‐line treatment for severe/very severe AA (SAA), in patients ≥ 50years, or transfusion‐dependent non‐severe AA (NSAA) includes immunosuppressive therapy (IST) with horse anti‐thymocyte globulin (hATG) and cyclosporine A (CSA) in combination with eltrombopag (EPAG; ACE‐scheme) [2].

During pregnancy, patients with AA frequently relapse or experience worsening of cytopenia, making management challenging, as current standard treatment is limited to blood product support owing to limited data on EPAG use in this setting [3, 4]. EPAG, a thrombopoietin receptor agonist, is classified as a pregnancy category C medication by the Food and Drug Administration due to the lack of controlled studies [4, 5]. Studies and case reports, particularly in pregnant patients with idiopathic thrombocytopenic purpura (ITP), highlighted potential adverse effects, including preeclampsia, low birth weight, and neonatal thrombocytopenia or thrombocytosis [4, 5].

## 2 Results

A 31‐year‐old female patient was referred to our department in August 2022 by a hematologist with the diagnosis of NSAA and transfusion dependency.

In late 2021, the patient consulted her family physician due to increasing hematomas. Laboratory tests revealed a pancytopenia (hemoglobin 9 g/dL, reticulocytes 61.3 × 10^9^/L, platelet count (PLC) 26 × 1^9^/L, absolute neutrophil count 1.65 × 10^9^/L). Bone marrow diagnostics in June 2022 showed a hypocellular bone marrow with reduced granulopoiesis, slightly impaired maturation of megakaryopoiesis and erythropoiesis, increased T‐lymphocytes, and marrow edema, indicating toxic‐immunological bone marrow damage. In addition, a PNH clone (granulocytes 7.8%) was detected using fluorescein‐labeled proaerolysin (FLAER). Consequently, NSAA was diagnosed in June 2022.

The patient was admitted to our hospital in October 2022 for IST with hATG, CSA, and EPAG due to persistent transfusion dependence, which was well tolerated.

**FIGURE 1:**
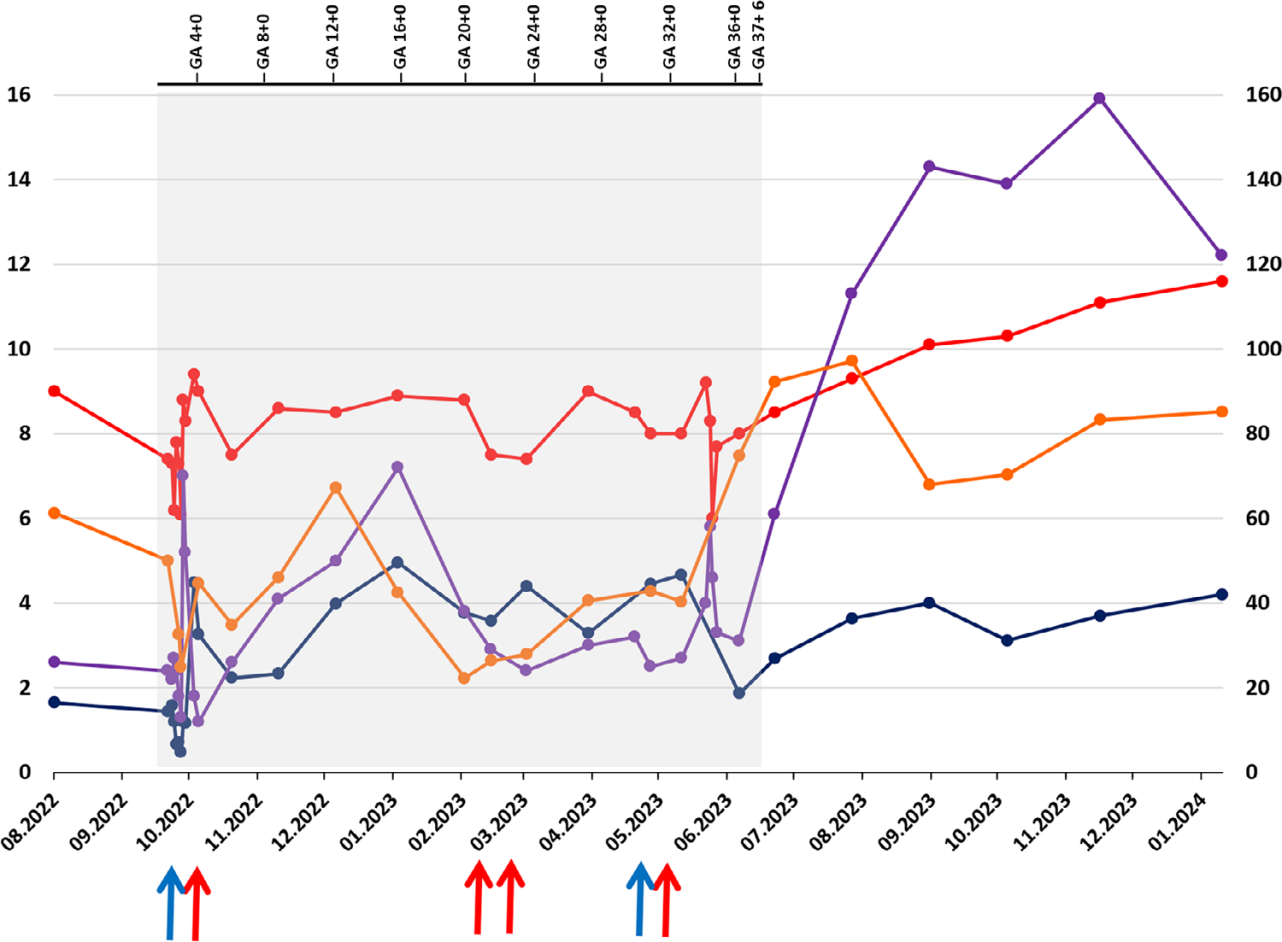
Course of blood values before and during therapy. Red line/dots: Hb in g/dL. Blue line/dots: ANC in ×10^9^/L. Violet line/dots: PLC in ×10^9^/L. Orange line/dots: Retic in ×10^9^/L. Blue arrows represent transfusions of platelets. Red arrows indicate packed red blood cell transfusions. Grey area pregnancy.

Outpatient follow‐up revealed no complications and the patient continued CSA and EPAG (150 mg/day). Based on gestational age calculations, conception is estimated to have occurred in mid‐October 2022. This was the first pregnancy with no history of miscarriage. After a risk‐benefit assessment together with the patient, therapy with full‐dose EPAG was continued, leading to reduced transfusion requirements. PLC remained stable (25–35 × 10^9^/L), with platelet transfusions required only prior to delivery. In the second trimester, she developed transfusion‐dependent anemia but maintained otherwise stable (Hb 8–9 g/dL; Figure 1). In February 2023, at 23 + 5 weeks of gestation, she tested positive for SARS‐CoV‐2 but remained asymptomatic, with only a mild AST and LDH elevation considered most consistent with hemolysis in the context of infection.

The further course of pregnancy was rather uneventful, with no signs of preeclampsia. Fetal growth was initially normal (58.7th percentile) until 34 + 0 weeks of gestation (estimated fetal weight 2465 g), but declined to the 18.4th percentile by 36 + 0 weeks of gestation, consistent with small for gestational age (SGA), and persisted until birth. Cesarean delivery was chosen based on the overall clinical situation and multidisciplinary assessment. At Week 37 + 6 of gestation, a cesarean section was performed without increased bleeding. The female neonate, in the 19th percentile (birth weight 2775 g; length 49 cm), was otherwise healthy (APGAR‐Score 6/8/9). Mild neonatal thrombocytopenia (126 × 10^9^/L) normalized to 175 × 10^9^/L within 1 week.

During outpatient follow‐up, the patient's PNH clone size declined to 4.7%, with persistence of transfusion independence. Interestingly, PNH clone size was smallest during pregnancy (3.7%). EPAG could be tapered (75 mg/day) in September 2023 and was discontinued in November 2023, followed by gradual CSA reduction. The patient continues to exhibit mild cytopenia (Hb 11.6 g/dL, PLC 122 × 10^9^/L, ANC 4.2 × 10^9^/L) under ongoing CSA but remains asymptomatic.

## 3 Discussion

AA typically worsens during pregnancy [3, 4]. Supportive care, primarily transfusions, is often insufficient due to disease progression; however remain the only therapeutic option during pregnancy [3, 4]. Although EPAG is not approved for use in pregnancy, several studies, particularly in ITP patients, demonstrate its efficacy in increasing maternal PLC while reporting relatively few adverse side effects [4, 5]. Complications such as preeclampsia, low birth weight, and neonatal thrombocytopenia have been reported, but it remains unclear whether these are due to EPAG or the underlying maternal disease (ITP, AA). Furthermore, maternal thrombocytopenia and transfusion dependence are associated with poorer pregnancy outcomes [4, 5].

Rottenstreich et al. reported that EPAG crosses the placenta in animals [5]. It is therefore expected that EPAG also crosses the placenta in humans, potentially causing neonatal thrombocytosis. However, only a few cases of thrombocytosis in newborns have been reported [5]. Neonatal thrombocytopenia is more frequently observed [4, 5]. In our case, the newborn exhibited mild thrombocytopenia (126 × 10^9^/L; RR 160–320 × 10^9^/L), whereas prior reports describe nadirs as low as 4 × 10^9^/L [5]. It remains unclear whether thrombocytopenia is directly caused by EPAG or if maternal conditions such as ITP or AA contribute to fetal platelet abnormalities.

This is the first reported case of a patient with NSAA who continued full‐dose EPAG (150 mg/d) through pregnancy. Two prior case reports describe a young female with AA receiving EPAG during pregnancy with variable outcomes. Suminaga et al. reported a patient on EPAG (50 mg/day) due to immunosuppressive‐refractory AA [4]. She discontinued therapy during the first 3 weeks of pregnancy due to hyperemesis gravidarum. Later, she developed preeclampsia and mild FGR [4]. Kawabata et al. described a patient with worsening cytopenias during pregnancy [6]. EPAG (25 mg/day) was initiated after Week 9 of gestation. Similar to Suminga's case, preeclampsia and FGR were observed [6]. Our patient received full‐dose EPAG (150 mg/day) throughout pregnancy, maintaining stable PLC (25–35 × 10^9^/L). She remained normotensive, and preeclampsia did not occur. Fetal growth was normal until week 34 + 0 of gestation (58.7th percentile), and an estimated fetal weight of 2465 g. Notably, all three reported neonates showed impaired fetal growth, presenting either with fetal growth restriction or being SGA at comparable gestational ages. There is evidence that neonates born to a patient with AA may have an increased risk to be SGA. Whether the observed growth impairment is attributable to EPAG exposure or to the underlying disease itself, namely AA, remains uncertain.

PNH frequently occurs with AA. Our patient had a PNH clone (7.8%), which declined to 3.7% during pregnancy and increased to 4.7% postpartum. Whether prior cases reported also had a PNH clone remains unknown, as this information was not mentioned [4, 6]. D‐dimer levels were elevated before pregnancy (6.97 mg/L) and rose further to 10.33 mg/L during pregnancy. No thrombotic events occurred, and thromboprophylaxis was withheld due to thrombocytopenia.

## 4 Conclusion

This is the first reported case of a pregnant patient with NSAA and PNH receiving full‐dose EPAG throughout pregnancy without toxicity. The pregnancy was uneventful, without preeclampsia, preterm delivery, or significant bleeding. The newborn exhibited mild thrombocytopenia and slight growth restriction in the final weeks, findings previously reported in AA pregnancies.

EPAG may be a viable treatment in patients with transfusion‐dependent AA during pregnancy, given careful risk‐benefit assessment. Further investigations are warranted to evaluate its safety and efficacy.

## Funding

The authors have nothing to report.

## Ethics Statement

This study was performed in accordance with the study protocol, the Declaration of Helsinki, and the International Council for Harmonization guidelines for Good Clinical Practice. All patients provided written informed consent for their participation in the study.

## Consent

The study was conducted in accordance with the Declaration of Helsinki. Written informed consent was obtained from the patient for publication of this case report and any accompanying images/details.

## Conflicts of Interest

Ferras Alashkar reports consultancy for Bristol Myers Squibb/Celgene, Global Blood Therapeutics/Pfizer, Novartis, and Vertex; honoraria from Agios, Bristol Myers Squibb/Celgene, Global Blood Therapeutics/Pfizer, Novartis, and Vertex; and research funding from Global Blood Therapeutics/Pfizer. H. Christian Reinhardt reports consultancy for Roche, Vertex, Janssen, and KinSea; honoraria from AbbVie, Aurikamed, Roche, Novartis, Takeda, and Amgen; research funding from Gilead and AstraZeneca; ownership interests in Fresenius, Roche, Novartis, Lilly, and Janssen; and is founder of CDL Therapeutics GmbH. Alexander Röth has received consultancy fees from Alexion Pharmaceuticals, Inc, Apellis Pharmaceuticals, Bioverativ, a Sanofi company, Novartis, Recordati, Roche, and Sanofi; honoraria from Alexion, Amgen, Apellis, Novartis, Recordati, Roche, Sanofi and Sobi, and advisory board fees from Alexion, Amgen, Apellis, Bioverativ, Novartis, Recordati, Roche, Sanofi, and Sobi. The other author declares no conflicts of interest.

## Data Availability

The data that support the findings of this study are available on request to the corresponding author.
